# Secreted frizzled-related protein 2 is epigenetically silenced and functions as a tumor suppressor in oral squamous cell carcinoma

**DOI:** 10.3892/mmr.2014.2542

**Published:** 2014-09-05

**Authors:** CAN XIAO, LILI WANG, LIFANG ZHU, CHENPING ZHANG, JIANHUA ZHOU

**Affiliations:** 1 Department of Occupational Medicine and Environmental Health, School of Public Health, Soochow University, Suzhou, Jiangsu 215123, P.R. China; 2 Department of Stomatology, The First Affiliated Hospital of Soochow University, Suzhou, Jiangsu 215006, P.R. China; 3 Department of Stomatology, Shanghai Ninth People’s Hospital, Shanghai Jiaotong University School of Medicine, Shanghai 200011, P.R. China

**Keywords:** oral squamous cell carcinoma, methylation, secreted frizzled-related protein 2, Wnt signaling pathway

## Abstract

The role of epigenetic inactivation of secreted frizzled-related protein 2 (SFRP2) and its functions in the development of oral squamous cell carcinoma (OSCC) remain to be elucidated. The present study demonstrated that SFRP2 mRNA was detected in 97.96% of tumor-adjacent normal tissues, while its expression was only detected in 16.33% of the tumor samples. In addition, the loss of SFRP2 expression was associated with hypermethylation of its promoter. As expected, the overexpression of SFRP2 in OSCC cell lines (Tca8113) suppressed cell proliferation and arrested the cell cycle in the G1 phase. Overexpression of SFRP2 also effectively repressed tumor growth in xenograft animals. Mechanistic investigations revealed that SFRP2 inhibited the development of OSCC *in vitro* and *in vivo* through an increase in the expression levels of glycogen synthase kinase-3β and a decrease in the expression level of cyclin D1, a direct read-out gene of active Wnt signaling. In addition, an increase in the expression of β-catenin was observed in the Tca8113/SFRP2 cells and in the animal models overexpressing SFRP2. Therefore, the results of the present study provide insight into the role of SFRP2 as a functional tumor suppressor in the development of OSCC through inhibition of the Wnt signaling pathway. Further studies on the precise mechanisms underlying the inhibition of Wnt signaling by SFRP2 and its association with β-catenin are required.

## Introduction

Oral squamous cell carcinoma (OSCC; ICD9 code 140-149, excluding 142 and 147) is the sixth most common human malignancy worldwide. Due to the high recurrence rate of primary tumors or the presence of a second primary tumor, the five-year survival rate of OSCC following diagnosis remains ~50% despite advances in surgical and radiotherapy treatment (1). Another important reason for this low survival rate is mainly due to the lack of understanding of the basic biological mechanisms involved in OSCC (2). In the last decade, epigenetic information, including DNA methylation, histone modification and chromatin structure has been demonstrated to be important in carcinogenesis (3,4). Similarly, several studies investigating DNA hypermethylation in head and neck cancer suggested that this phenomenon may be critical in OSCC development (5,6).

The Wnt signaling pathway is involved in numerous developmental processes and in tumorigenesis (7,8). Usually, the binding of Wnt to frizzled receptors leads to cytosolic stabilization and accumulation of β-catenin, which translocates into the nucleus and interacts with TCF/LEF transcription factors thereby regulating the expression of downstream target genes, including cyclin D1. The Wnt signaling pathway can be partially regulated by Wnt antagonists, including members of the Dickkopf family, Wnt inhibitory factor 1 and secreted frizzled-related proteins (SFRPs). The SFRPs are a family composed of five soluble glycoproteins (SFRP1-5), each containing a cysteine-rich domain. These domains are homologous to the putative Wnt-binding sites of frizzled proteins. Therefore, SFRPs may prevent frizzled receptors from binding to Wnt proteins and, in turn, downregulate Wnt signaling.

A previous study confirmed that the epigenetic inactivation of SFRP2, a member of the SFRP family, is important in the progression of several types of human tumor, including head, colorectal and breast cancer (9–11). However, few studies have been performed to investigate the functional significance of SFRP2 in the development of OSCC. Based on analysis of the SFRP2 promoter methylation status and the expression levels of SFRP2 in cancer tissues and adjacent non-cancer tissues from OSCC patients, the present study investigated the effects of SFRP2 on the Wnt signaling pathway in the development of OSCC *in vivo* and *in vitro*.

## Materials and methods

### Human OSCC samples

A total of 49 cancer tissues and their adjacent non-cancer specimens were obtained from patients diagnosed with OSCC, who had undergone curative surgery at the Department of Oral and Maxillofacial Surgery, The First Affiliated Hospital of Soochow University, (Suzhou, China). The collected specimens were immediately snap-frozen in liquid nitrogen and stored at −196°C for further analysis. The present study was approved by the Clinical Research Ethics Committee of Soochow University and all patients provided written informed consent for obtaining the study materials.

### Cell lines and cell culture

The human tongue squamous cell carcinoma cell line Tca8113 was obtained from the China Center for Type Culture Collection (Shanghai, China). Cells were cultured in RPMI-1640 containing 10% fetal bovine serum (Invitrogen Life Technologies, Carlsbad, CA, USA) at 37°C in 5% CO_2_ and 95% humidity.

### DNA and RNA extraction

Genomic DNA was extracted from the cancer tissues and their adjacent non-cancer specimens using a DNA mini kit (Qiagen, Hilden, Germany) according to the manufacturer’s instructions. The DNA was subsequently resuspended in 500 μl LoTE (2.5 mmol/l EDTA and 10 mmol/l Tris-HCl; MayBiotech, Nanjing, China) and stored at −80°C until use.

Total RNA was extracted from 49 pairs of tissue specimens and Tca8113 cells using TRIzol reagent (Invitrogen Life Technologies) according to the manufacturer’s instructions. The RNA was resuspended in 500 μl LoTE buffer and stored at −80°C until use.

### Methylation-specific polymerase chain reaction (MSP)

The methylation status of the SFRP2 promoter in the OSCC tissues and Tca8113 cells was determined by MSP. Briefly, 2 mg genomic DNA was bisulphite-treated using a Zymo DNA Modification kit (Zymo Research, Orange, CA, USA) and was then used as a template for MSP. The MSP conditions were as follows: One cycle at 95°C for 3 min followed by 30 cycles of 95°C for 30 sec, 53.5°C for 30 sec and 72°C for 1 min. The detection of methylation included use of a methylated (M) reaction and an unmethylated (U) reaction. The primer sequences used were as follows: M, forward 5′-TTTTTACGGTATTGGGGAGTATATC-3′ and reverse 5′-AAAAACCAATAAAAAATAATCCGA-3′; U, forward 5′-TTATGGTATTGGGGAGTATATTGA-3′ and reverse 5′-AAAACCAATAAAAAATAA TCCAAA-3′.

### Reverse transcription polymerase chain reaction (RT-PCR)

A reverse transcription reaction was performed using 2 mg total RNA using a first strand cDNA kit (Roche Diagnostics, Mannheim, Germany). The mRNA expression of SFRP2 was detected by RT-PCR and GAPDH was used as an internal control of RNA integrity. The primer sequences used were as follows: SFRP2, forward 5′-TGGAGACCAAGAGCAAGAC-3′ and reverse 5′-GTGGGACAAAGACAGGGTA-3′; GADPH, forward 5′-GAAGGTGAAGGTCGGAGTC-3′ and reverse 5′-GAAGATGGTG ATGGGATTTC-3′.

### Plasmid construction and generation of stable SFRP2 cell lines

A human SFRP2 expression vector, pcDNA3.1/SFRP2, was constructed by subcloning the full-length cDNA of SFRP2 into the *Hin*dIII-XhoI site of the pcDNA3.1(+) vector (Invitrogen Life Technologies). Following confirmation by sequencing, the pcDNA3.1/SFRP2 and pcDNA3.1 (control) were transfected into Tca8113 cells using FuGENE^®^ HD (Roche Diagnostics) according to the manufacturer’s instructions. Using 500 μg/ml G418 (Invitrogen Life Technologies) and confirming with RT-PCR and western blot analysis, single colonies of transfected cells exhibiting stable SFRP2 expression were selected for generation.

### Cell cycle analysis

The cell cycle was analyzed using flow cytometry (Cell Lab Quanta SC; Beckman Coulter, Miami, FL, USA) with 4′,6-diamidino-2-phenylindole staining (Sigma-Aldrich, St. Louis, MO, USA), as described in a previous study (12). Measurements were repeated independently three times.

### In vivo tumor growth

Suspensions of the stable SFRP2-expressing cells and the control cells (1×10^7^ in 200 μl RPMI-1640) were injected subcutaneously into 4–5-week old female nude mice (strain BALB/c nu/nu). Tumor size was measured using calipers every 3 days for a total of 4 weeks and tumor volume was calculated on the basis of width (x) and length (y) using the following formula: x2y / 2, where x<y.

### Western blot analysis

The protein extracted from the tissues and cells was resolved by SDS-PAGE and transferred onto polyvinylidene fluoride membranes (Hybond-P; cat no. 10600088; GE Healthcare, Buckinghamshire, UK) followed by western blot analysis. Signals were detected using an enhanced chemiluminescence detection system (ECL Plus Western Blotting Detection system; GE Healthcare). Primary goat anti-SFRP2 antibody was purchased from Sigma-Aldrich (cat: SAB2500934; Shanghai, China). Goat antibodies against glycogen synthase kinase 3β (GSK-3β; sc-8257) and β-catenin (sc-1496), and rabbit antibodies against cyclin D1 (sc-753) and β-actin (sc-130656) were purchased from Santa Cruz Biotechnology, Inc. (Santa Cruz, CA, USA). The poly-HRP anti-goat IgG was purchased from Abcam (ab6741; Cambridge, MA, USA). The goat anti-rabbit IgG (NA934) were purchased from GE Healthcare.

### Immunohistochemistry

The tissue sections were deparaffinized in xylene and dehydrated in ethanol (Sigma-Aldrich). Following dehydration, endogenous peroxides were inhibited, the sections were incubated with the goat polyclonal GSK-3β (sc8257), β-catenin (sc-1496) and rabbit polyclonal cyclin D1 (sc-753) primary antibodies and poly-HRP anti-goat (ab6741) or anti-rabbit IgG (ab6721; Abcam) secondary antibodies, and then incubated with 3,3′-diaminobenzidine (Sigma-Aldrich). The sections were then counterstained and dehydrated. Antibodies against GSK-3β, β-catenin and cyclin D1 were purchased from Santa Cruz Biotechnology, Inc.

### Statistical analysis

The SPSS software program for Windows (version 13; SPSS, Inc., Chicago, IL, USA) was used for statistical analysis. The association between methylation of the SFRP genes and the clinical parameters were analyzed using a χ^2^ test and a Fisher’s exact test, where necessary. P<0.05 was considered to indicate a statistically significant difference.

## Results

### Methylation status of the SFRP2 promoter in OSCC tissues

Through MSP, the methylation status of the SFRP2 promoter was evaluated in 49 cases of OSCC and corresponding normal tumor-adjacent tissues. The methylation of the SFRP2 promoter was detected in 37 tumor samples (75.51%). However, in the corresponding normal tumor-adjacent tissues, the SFRP2 promoter was methylated in only three cases (6.12%). The frequency of SFRP2 promoter methylation in OSCC tissues was significantly higher than that in the adjacent tissues (χ^2^=46.00; P<0.001).

### Expression of SFRP2 mRNA in OSCC tissues

The present study detected the SFRP2 mRNA expression in 49 sample pairs of OSCC and their adjacent non-cancerous tissues by quantitative RT-PCR (Data not shown). SFRP2 mRNA was detectable in 48 corresponding normal tumor-adjacent tissues (97.96%). However, of the 49 OSCC tissues, only eight samples exhibited SFRP2 mRNA expression (χ^2^=63.37; P<0.001). Furthermore, in 35 (94.59%) of the 37 OSCC tissue samples demonstrating methylation of the SFRP2 promoter, there was no detectable expression of SFRP2 mRNA. In the 12 OSCC tissue samples that did not demonstrate methylation of the SFRP2 promoter sequence, SFRP2 mRNA was detected in six samples (χ^2^=10.13; P<0.01) (Data not shown).

### SFRP2 inhibits OSCC cell proliferation

**Figure 1:**
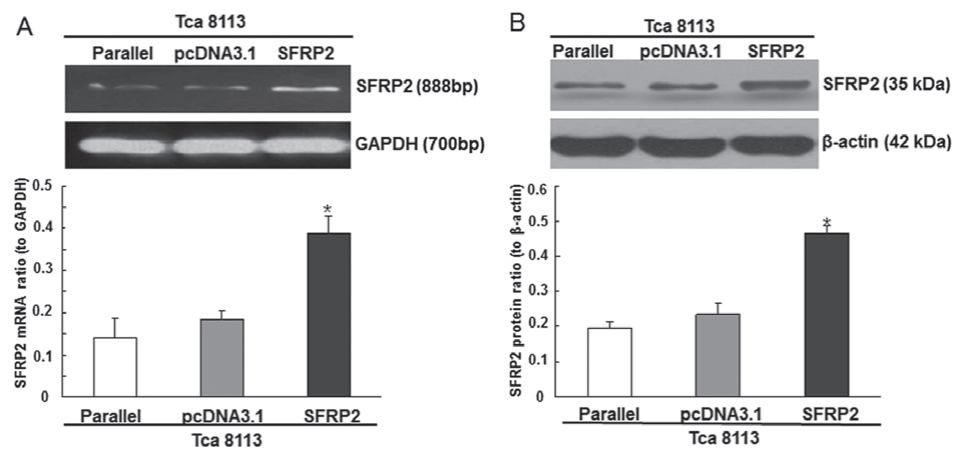
Expression of (A) SFRP2 mRNA and (B) SFRP2 protein in different cell lines. Parallel: Tca 8113 cells; pcDNA3.1: Tca 8113 cells transfected with pcDNA3.1; SFRP2: Tca 8113 cells transfected with pcDNA3.1/SFRP2. SFRP2, secreted frizzled-related protein 2.

The frequent silencing of SFRP2 by methylation in OSCC, but not in non-cancerous tissues suggests SFRP2 has a potential tumor-suppressing effect in the development of OSCC. To assess this hypothesis, the present study examined the effect of SFRP2 overexpression on the proliferation and apoptosis of Tca8113 cells. Compared with the Tca8113 cells transfected with pcDNA3.1, the cells transfected with the pcDNA3.1/SFRP2 plasmids demonstrated increased expression levels of SFRP2 (Fig. 1A and B). In addition, 48 h after transfection, overexpression of SFRP2 significantly inhibited the proliferation of Tca8113 cells and arrested the cell cycle in the G1 phase (Table I and II).

### Effects of SFRP2 on the Wnt signaling pathway in OSCC cells

**Figure 2:**
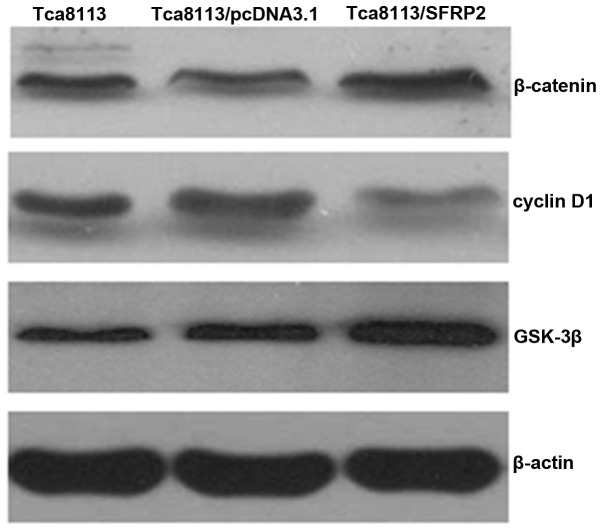
Expression levels of GSK-3β, β-catenin and cyclin D1 in Tca8113, Tca8113/pcDNA3.1 and Tca8113/SFRP2 cells. Tca 8113/pcDNA3.1: Tca 8113 cells transfected with pcDNA3.1; Tca 8113/SFRP2: Tca 8113 cells transfected with pcDNA3.1/SFRP2. SFRP2, secreted frizzled-related protein 2; GSK-3β, glycogen synthase kinase-3β.

To further understand the effects of SFRP2 on the Wnt signaling pathway in OSCC, the expression levels of GSK-3β, β-catenin and cyclin D1 were analyzed in the Tca8113 cells, Tca8113/pcDNA3.1 cells and Tca8113/SFRP2 cells, respectively. Compared with the parental and mock-transfected control cells, increased levels of GSK-3β and β-catenin were observed in the cells exhibiting SFRP2 overexpression. In addition, the growth promoting gene cyclin D1, a direct read-out gene of the active Wnt signaling pathway, was significantly decreased in the Tca8113/SFRP2 cells (Fig. 2).

### SFRP2 inhibits OSCC growth in vivo

**Figure 3:**
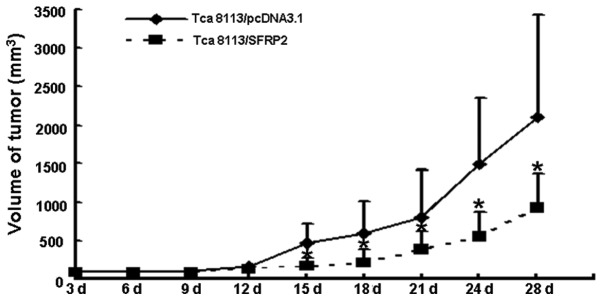
Tumor growth curves of the animals inoculated with Tca8113/pcDNA3.1 and Tca8113/SFRP2 cells, respectively. Tca8113/pcDNA3.1: animals inoculated with Tca8113/pcDNA3.1; Tca8113/SFRP2: animals inoculated with Tca8113/SFRP2. SFRP2, secreted frizzled-related protein 2.

Following the observed anti-proliferative effects of SFRP2 on Tca8113 cells, the present study then assessed whether SFRP2 inhibits the growth of OSCC *in vivo*. Following subcutaneous inoculation with Tca8113/pcDNA3.1 cells and Tca8113/SFRP2 cells, all the animals developed OSCC and exhibited the typical histopathological characteristics of human OSCC. However, as indicated by the tumor growth curve, the mean tumor volume was significantly smaller in the SFRP2-transfected nude mice than that in the animals inoculated with the Tca8113/pcDNA3.1 control (P<0.001; Fig. 3).

### Effects of SFRP2 on the Wnt signaling pathway in vivo

**Figure 4:**
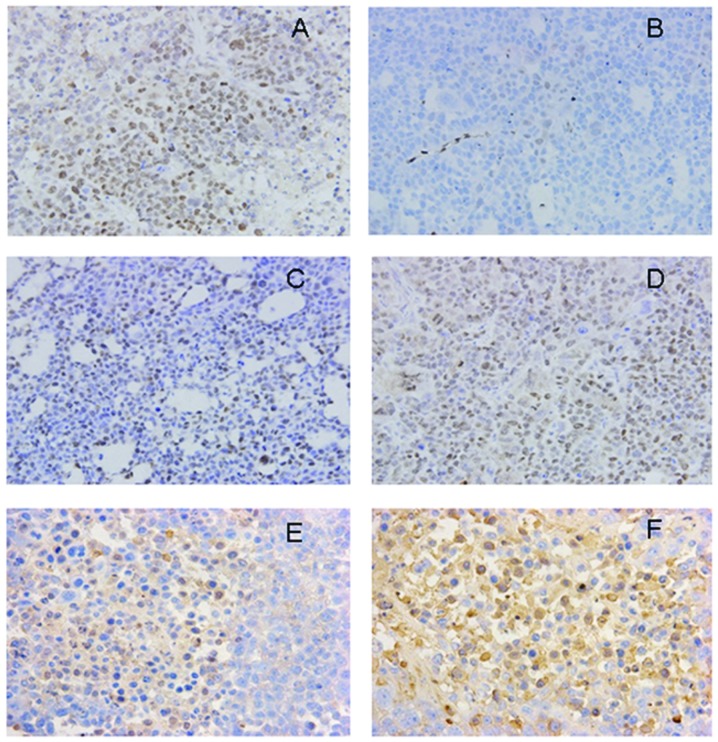
Immunohistochemical analysis of the expression levels of cyclin D1, glycogen synthase kinase-3β and β-catenin in representative samples from the animals inoculated with Tca8113/pcDNA3.1 and Tca8113/SFRP2 cells (stained by 3,3′-diaminobenzidine). (A, C and E) Tissues from the group of animals inoculated with Tca8113/pcDNA3.1. (B, D and F) Tissues from the group of animals inoculated with Tca8113/SFRP2. (magnification, ×200). SFRP2, secreted frizzled-related protein 2.

In order to investigate whether SFRP2 affects the growth of OSCC via the Wnt signaling pathway in nude mice, the expression levels of GSK-3β, β-catenin and cyclin D1 in tumor tissues were further analyzed by immunohistochemical analysis. Compared with the animals in the Tca8113/pcDNA3.1 group, the expression level of cyclin D1 was markedly decreased (Fig. 4), while the expression levels of GSK-3β and β-catenin was significantly increased in the cell cytoplasm and cell membrane in the animals from the Tca8113/SFRP2 group (Fig. 4).

## Discussion

Several previous studies indicated that hypermethylation of a gene promoter is critical in silencing tumor suppressor genes in various types of human malignancy, including breast cancer, oral cancer and head and neck cancer (13–15). The SFRP gene family, which functionally act as Wnt signaling inhibitors, are a common target of promoter hypermethylation in several types of tumor (16,17). Similar to these observations, the results of the present study demonstrated that methylation of the SFRP2 promoter in OSCC tissues occurred more frequently than in the normal tumor-adjacent tissues (75.51 vs. 6.12%). As a result, the SFRP2 mRNA expression was significantly inhibited in the OSCC tissue samples. In addition, in the OSCC tissue samples exhibiting SFRP2 promoter methylation, the mRNA expression of SFRP2 was markedly decreased compared with those without SFRP2 promoter methylation. Therefore, the results of the present study indicated that SFRP2 promoter methylation was an important mechanism in the development of OSCC and suggested that downregulation in the expression of SFRP2 is likely to be due to methylation of the SFRP2 promoter.

The Wnt signaling pathway is known to be involved in tumorigenesis in several types of human cancer (18,19). The identification of Wnt signaling pathway antagonists, including SFRPs family members, signified a new era in the study of the Wnt gene and its effects (20–22). Similar to other members of the SFRP family, SFRP2 is able to negatively regulate the Wnt signal transduction pathway (11). Overexpression of SFRP2 generally causes suppression of the Wnt signaling pathway and inhibition of cell proliferation (23,24). In the present study, the overexpression of SFRP2 significantly inhibited Tca8113 cell proliferation and arrested the cell cycle in the G1 phase. Additionally, the growth promoting gene cyclin D1, a direct read-out gene of the active Wnt signaling pathway, was significantly decreased in the cells overexpressing SFRP2. In addition, using an OSCC nude mice model, the present study further confirmed that SFRP2 inhibited the growth of OSCC *in vivo*. The expression level of cyclin D1 was also decreased significantly, while GSK-3β, an inactivated signal protein of the Wnt signaling pathway, was increased significantly in the animals with SFRP2 overexpression. Therefore, these findings suggested that methylation of the SFRP2 gene and the consequent regulation of protein expression may be involved in the development of OSCC and is likely to be associated with overactivation of the Wnt signaling pathway.

β-catenin is a multifunctional protein involved in two independent processes, including cell-cell adhesion and signal transduction (25). In addition to its effect on the regulation of cell adhesion, β-catenin is a key effector in the Wnt signaling pathway (26,27). When β-catenin accumulates in the cytoplasm and moves into the nucleus of the cell, it can activate the Wnt signaling pathway and accelerate the progress of cancer. As expected, accumulation of β-catenin was detected in the cytoplasm of OSCC cells in the present study. However, although Tca8113 cell proliferation and OSCC development were inhibited by the overexpression of SFRP2, increased expression levels of β-catenin were unexpectedly observed in the Tca8113/SFRP2 cells and in the animal models. The underlying mechanisms causing these increased levels of β-catenin and the biological functions of the increased expression of β-catenin remain to be elucidated. In previous studies, various patterns of β-catenin expression have been observed in human cancer tissues (28). For example, it was reported that 88% of head and neck squamous cell carcinomas exhibited reduced β-catenin expression (29,30). By contrast, increased levels of β-catenin were detected in several types of tumor, including liver cancer and lung cancer (31,32). Thus, in order to clarify the biological functions of β-catenin and its association with SFRP2 in OSCC development, more detailed studies are required in the future.

Taken together, the data from the present study demonstrated that hypermethylation of the SFRP2 promoter is important in the development of OSCC *in vivo* and *in vitro*. In addition, by increasing the expression of GSK-3β and decreasing the expression of cyclin D1, SFRP2 had the ability to inactivate the Wnt signaling pathway in OSCC development. Further understanding of the precise mechanisms of how SFRP2 inhibits the Wnt signaling pathway and its association with β-catenin is important for improving the design of anticancer strategies against OSCC.

## Figures and Tables

**Table I:** Effects of SFRP2 overexpression on the proliferation of Tca8113, Tca8113/pcDNA3.1 and Tca8113/SFRP2 cells.

	OD570 nm (MTT)			
	
Group	Day 1	Day 2	Day 3	Day 4
Tca8113	0.3667±0.0349	0.6358±0.0295	1.0427±0.1087	1.0915±0.1536
Tca8113/pcDNA3.1	0.3962±0.0451	0.6351±0.1865	1.0338±0.1265	1.1217±0.2294
Tca8113/SERP2	0.3342±0.0667	0.6621±0.2333	0.6961±0.1661^a^	0.8041±0.0277^a^

a P<0.05, compared with Tca8113 and Tca8113/pcDNA3.1.

SFRP2, secreted frizzled-related protein 2.

**Table II:** Effects of SFRP2 overexpression on the cell cycle distribution of Tca8113, Tca8113/pcDNA3.1 and Tca8113/SFRP2 cells.

	Cell cycle distribution (%)		
	
Group	G1	G2	S
Tca8113	46.839±1.516	17.431±0.989	33.265±1.604
Tca8113/pcDNA3.1	48.472±1.879	16.748±1.133	34.780±1.416
Tca8113/SFRP2	56.433±1.348^a^	11.758±2.839^a^	31.809±1.537

a P<0.05, compared with Tca8113 and Tca8113/pcDNA3.1.

SFRP2, secreted frizzled-related protein 2.
